# Influence of electronic nicotine delivery systems (ENDS) in comparison to conventional cigarette on color stability of dental restorative materials

**DOI:** 10.12669/pjms.36.5.2303

**Published:** 2020

**Authors:** Fahim Vohra, Abdulelah F. Andejani, Osamah Alamri, Abdulaziz Alshehri, Rana S Al-Hamdan, Thamer Almohareb, Tariq Abduljabbar

**Affiliations:** 1 Fahim Vohra Department of Prosthetic Dental Science, College of Dentistry, King Saud University, Riyadh 11545, Saudi Arabia; 2 Abdulelah F Andejani, Department of General Dentistry, College of Dentistry, King Saud University, Riyadh 11545, Saudi Arabia; 3 Osamah Alamri Department of General Dentistry, College of Dentistry, King Saud University, Riyadh 11545, Saudi Arabia; 4 Abdulaziz Alshehri Department of General Dentistry, College of Dentistry, King Saud University, Riyadh 11545, Saudi Arabia; 5 Rana S Al-Hamdan Department of Restorative Dental Sciences, College of Dentistry, King Saud University, Riyadh 11545, Saudi Arabia; 6 Thamer Almohareb Department of Restorative Dental Sciences, College of Dentistry, King Saud University, Riyadh 11545, Saudi Arabia; 7 Tariq Abduljabbar Department of Prosthetic Dental Science, College of Dentistry, King Saud University, Riyadh 11545, Saudi Arabia

**Keywords:** Ceramic, Cigarette, Color stability, Electronic nicotine delivery system, Resin composite

## Abstract

**Objective:**

The use of Electronic Nicotine Delivery Systems (ENDS) is increasing rapidly. However, its discoloring effect on dental restorations is not known. This study aimed to evaluate the effect of ENDS aerosol when compared to conventional cigarette smoke (CS) on the color stability of dental ceramic (DC) and resin composite (RC).

**Methods:**

This research project was conducted from November 2018 to May 2019. In this study 30 discs each for DC and RC materials were fabricated to be equally divided into groups of exposure to CS, ENDS aerosol and storage in distilled water (No smoke; NS) respectively (n=10). Specimens were exposed for a total of 7 days, with a rate of 10 cycles per day, each cycle represented 10 puffs. The color change was assessed using the CIELAB color space, by calculating ΔE. Data was analysed using ANOVA and multiple comparisons test.

**Results:**

Ceramic specimens in CS (2.422 ± 0.771) and ENDS (2.396 ± 0.396) groups showed comparable ΔE (color change) (p=0.992). Similarly, composite specimens in CS (42.871 ± 2.442) and ENDS (46.866 ± 3.64) groups showed comparable ΔE (p>0.05). NS specimens in both composite and ceramic samples showed lower ΔE than CS and ENDS specimens respectively.

**Conclusions:**

Aerosol from Electronic nicotine delivery systems (ENDS) showed similar discoloration levels as cigarette smoking (CS). The level of discoloration for ceramic samples for both ENDS and CS was below clinically perceptible levels (Mean ΔE < 2.5). Discoloration of composite resin due to CS and ENDS was visually perceptible (Mean ΔE > 4.0).

## INTRODUCTION

The demand for cosmetic dental therapy is increasing and more individuals are seeking an ideal smile.^1^ Tooth colored restorations are commonly provided in the form of adhesive composite resin and dental ceramics. Improved dental composites are minimally invasive, preserve tooth structure, are mechanically robust (high abrasion resistance and compressive strength) with excellent longevity and show acceptable translucency.^2^ Bondable glass ceramics are commonly used for esthetic veneers and lumineers, for smile enhancement with improved tooth shade, translucency and low intra-oral degradation.^3^ Esthetic outcomes and their longevity is influenced by surface characterization, surface gloss, oral hygiene maintenance and exposure to intrinsic and extrinsic stains.^3^ With developments in esthetic restorative materials, color stability is critical in selection of these materials and extrinsic staines are commonly associated with esthetic compromise.^1^

Sources of extrinsic stains include plaque, oral pigments, carbonated beverages, coffee, tea and cigarette smoking.^1^ Cigarette smoking is by far the most common cause of superficial dental staining, with more than 1.3 billion smokers around the world.^4^ Staining from cigarette is attributed to the brown pigment from leaves of tobacco in particulate form called tar.^5^ Smokers are self-aware of increased perceived dental discoloration and smile dissatisfaction as compared to non-smokers as shown in a previous study.^6^ Resistance to restorative discoloration is challenging in the presence of cigarette smoking; and techniques including the use of abrasives for physical stain removal with polishing and chemical bleaching agents are employed for regaining esthetics.

Electronic Nicotine Delivery Systems (ENDS) also known as e-cigarettes or vapes, were introduced as an alternate to cigarette smoking in an effort towards smoking cessation.^7^ ENDS is an electronic device, which heats a liquid with different nicotine content and flavouring agents. The heated liquid vaporizes without combustion into an aerosol inhaled by the smoker, therefore generating few harmful chemical compounds in contrast to cigarette smoking.^8^ However the oxidation of e-liquid in ENDS releases large concentration of carbonyl compounds and metal oxides, with little evidence of their toxic effects on oral health.^9^ The contents of ENDS shows wide variations, therefore its effect on general and oral health are inconsistent.^9^ The proportion of ENDS users to conventional cigarette smokers has increased in recent years, suggesting that more smokers are switching to e-cigarettes.^10^

A recent study on the influence of vaping on the color change of teeth concluded, that ENDS aerosol discolored teeth above clinically perceptible levels.^11^ However, evidence related to the influence of ENDS on discoloration of dental restorative materials is lacking. As cigarette smoking causes the release of pigments from combustion, it is hypothesized that resin composite and dental ceramics when exposed to aerosol produced from oxidation of e-liquid (ENDS), will show better color stability in comparison to cigarette smoke exposure. Therefore, the aim of the study was to evaluate the effect of ENDS aerosol when compared to conventional cigarette smoke (CS) on the color stability of lithium disilicate ceramic and resin composite materials.

## METHODS

This research project protocol was reviewed and approved by the Research and ethics committee of College of dentistry, King Saud University with project no. FR-0411. The Project was conducted at College of Dentistry, King Saud University between November 2018 to May 2019. The in-vitro experiment was performed according to the checklist for reporting in-vitro studies (CRIS) guidelines.

### Exposure Products and Materials

The color stability of two groups of restorative materials were tested in this study; Resin Composite- RC (CoreRestore2™, Untinted – Kerr) and Dental Ceramic DC (IPS Empress Esthetic, E TC1 – Ivoclar Vivadent). Commercial tobacco cigarettes (Marlboro Gold–Philip Morris) and Electronic Nicotine Delivery System (SMOK - Alien 220w / used at 39.9 W, 4.1 volts, 28°-36° centigrade) were used to create exposure for CS and ENDS groups, respectively. E-liquid (Vapecrib – Cloudniners Mango; PG/VG: 30/70, Nicotine strength: 3mg) and EC coils were changed following daily exposure.

### Specimens Preparation

Each of the two groups, RC and DC contained 30 disk specimens, which were divided into 3 subgroups (n=10); conventional cigarette smoke (CS), electronic nicotine delivery systems smoke (ENDS) and no smoke (NS) groups. All specimens were prepared into disks of 10mm diameter and 2mm thickness. RC specimens were fabricated using a matrix, a polyester strip and bulk-filled RC cured with a dental light unit (Bluephase ® C8, Ivoclar Vivadent, Schaan, Liechenstein-650 mWcm-²) for 40 seconds. RC specimens were finished and flattened on a grinding unit (Pacer Industries Inc, PA USA). For the DC group disc specimens, Wax-up of discs was fabrication (10mm x 2mm) and invested. Using hot press technique, ceramic ingots were pressed at 920° C in press furnace. All specimens were cleaned in an ultrasonic bath with distilled water for 5 minutes and stored for 24 hours.

### Specimen Exposure

Specimens were allocated randomly to CS, ENDS and NS groups (n=10). This resulted in six study groups namely-

1. Resin Composite-Cigarette smoking. RC-CS.
2. Resin composite- Electronic Nicotine Delivery System. RC-ENDS.
3. Resin composite- No smoke. RC-NS.
4. Dental ceramic- Cigarette smoking. DC-CS.
5. Dental ceramic-Electronic Nicotine Delivery System. DC-ENDS.
6. Dental ceramic- No smoke. DC-NS.

**Fig.1:**
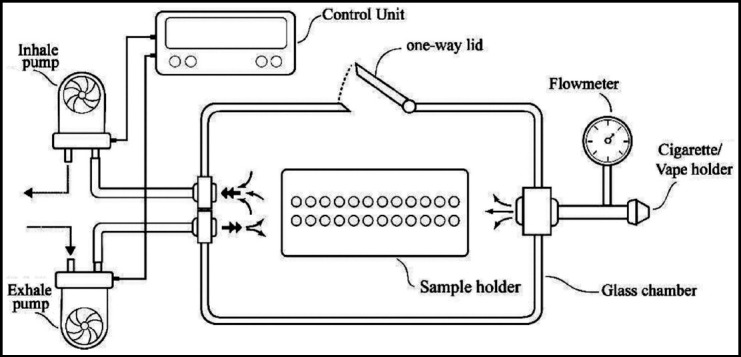
Smoke chamber designed and used for specimen exposure to electronic nicotine delivery system (ENDS) aerosol and cigarette smoke.

A custom-made smoking chamber in the form of a glass box of 10x10x5cm dimensions was used (Fig.1). The chamber had a lid, which provided a hermetic seal; and to act as an inlet and outlet for smoke, two vertically aligned vents were cut through one wall, the holes were located at the middle of the wall. Tubes were inserted into each hole, which was then sealed. The ends of the tubes were connected to a vacuum. A one-way valve was placed on the top wall, opening at high pressure to allow the smoke to escape after the preset duration of exposure. The vacuum system had two pumps, creating negative pressure inside the chamber to force smoke in, and positive pressure to force smoke out. Pumps were controlled by timers to turn on and off for a specified duration. A flow equivalent to 30cm^3^/s was created, and a flow-meter monitored the inward and outward flow. A cigarette holder was placed on a wall allowing cigarettes to be held horizontally, and permitting vertical placement of the electronic cigarettes.

Exposure parameters were standardized between the CS group and ENDS specimens which were exposed for a total of 7 days, with a rate of 10 cycles per day, each cycle represented 10 puffs, with a two seconds puff duration and a one puff every 30 seconds, repeatedly. Both ENDS and CS groups received similar amount of smoke exposure. NS group was stored in artificial saliva at 37°C for the same time period.

### Color Analysis

All specimens were stored in artificial saliva at 37° C for 24 hours prior to obtaining baseline color reading. A pre-exposure color analysis was done using a Spectrophotometer (Crystaleye; Olympus, Tokyo, Japan) according to CIELAB color space,^12^ which uses the color coordinates L*, a* and b* in a specific formula to calculate the total color change in three axes as ΔE. “L”, which is the lightness of the object is calculated from (0) black to (100) white. “a”, characterizes red to green coordinates (-90 to 70) and “b” assesses yellow to blue axes (-80 to 100).

Specimens were cleaned from dust mounted on the platform with a customized holder to standardize orientation and placement at 45° angle to the tip. The spectrophotometer, was re-calibrated before each measurement focusing on a 2mm diameter area of specimen. There measurements were recorded for each specimen before smoke exposure and a mean was identified. After the experiment, color analysis was performed the same way as the baseline. The color difference (ΔE) among the baseline and post exposure specimens was calculated by comparing ΔL, Δa and Δb and an overall ΔE using the following equation:

ΔE = [(ΔL)^2^ + (Δ a)^2^ + (Δb)^2^]1/2^12^

### Statistical Analysis

Means and standard deviations were identified and compared among study groups using Analysis of Variance (ANOVA) and Tukey multiple comparisons test (SPSS v10; SPSS Inc., Chicago, IL, USA). A p value of <0.05 was considered statitically significant.

## RESULTS

The means and standard deviations for ΔL* (L1-L2), Δa* (a1-a2) and Δb* (b1-b2) for ceramic and composite specimens among study groups are presented in Table-I and Table-II. The color difference (ΔE) value for each study group was identified by incorporating ΔL*, Δa* and Δb* in the equation.

**Table-I:** Mean and standard deviations for CIELab values and ΔE at 95% confidence interval among ceramic study groups.

Ceramic Study groups	ΔL* Mean (SD)	Δa* Mean (SD)	Δb* Mean (SD)	ΔE* Mean (SD)
Cigarette smoke (CS)	-2.108 (0.628)	0.331 (0.174)	1.12 (0.493)	2.422 (0.771) ^A^
E-cigarette smoke (ENDS)	-2.067 (0.389)	-0.579 (0.263)	1.137 (0.398)	2.396 (0.596) ^A^
No Smoke (NS)	-0.111 (0.347)	0.027 (0.032)	0.025 (0.073)	0.291 (0.23) ^B^

Dissimilar superscript alphabets for ΔE indicate statistical significance, SD. Standard deviation. ΔE values: NS < ECS = CS.

**Table-II:** Mean and standard deviations for CIELab values and ΔE at 95% confidence interval among composite study groups.

Composite Study groups	ΔL* Mean (SD)	Δa* Mean (SD)	Δb* Mean (SD)	ΔE* Mean (SD)
Cigarette smoke (CS)	-41.346 (2.407)	-6.867 (1.283)	-9.001 (1.305)	42.871 (2.448)^A^
E-cigarette smoke (ENDS)	-44.377 (3.637)	-5.894 (1.729)	-13.81 (1.863)	46.866 (3.641) ^A^
No Smoke (NS)	0.519 (0.337)	0.052 (0.12)	0.041 (0.143)	0.558 (0.329) ^B^

Dissimilar superscript alphabets for ΔE indicate statistical significance, SD. Standard deviation. ΔE values: NS < ECS = CS.

Among the ceramic specimens, the highest ΔE was observed in CS (2.422 ± 0.771) specimens, however the lowest ΔE was shown by control specimens (NS) (0.291 ± 0.23) (Table-I). Overall, specimens among ceramic study groups showed significant color difference (ΔE) (ANOVA, p<0.05). Statistical analysis showed comparable (p=0.992) ΔE (color change) among the CS (2.422 ± 0.771) and ENDS (2.396 ± 0.396) specimens. Specimens in the NS group showed lower ΔE values in comparison to ENDS and CS specimens, which was significantly different (p=0.001).

Among the composite specimens, the maximum ΔE was shown by specimens exposed to ENDS aerosol (46.866 ± 3.64) (Table-II). However control specimens stored in distilled water and not exposed to smoke (NS) showed minimum ΔE (0.558 ± 0.32) among tested specimens. Overall, specimens among composite study groups showed significant color difference (ΔE) (ANOVA, p<0.05). ΔE among the specimens exposed to cigarette smoke (CS) (42.871 ± 2.44) was lower than specimens exposed to ENDS aerosol (46.866 ± 3.64), however they were statistically comparable (p>0.05). Specimens among the control composite group (NS) showed significantly lower ΔE compared to ENDS and CS specimens respectively (p<0.05).

## DISCUSSION

The present study was based on the hypothesis that resin composite and dental ceramics when exposed to aerosol produced from oxidation of e-liquid, will show better color stability in comparison to cigarette smoke exposure. The experiment outcomes showed that the discoloration caused by ENDS aerosol was similar to CS among comparable groups of composite and ceramic specimens, therefore the hypothesis was rejected. A major cause of the discoloring effect of ENDS aerosol is attributed to the release of metal ions, pigments and particles on heating of the e-liquid.

It is known that cigarette smoking is a cause of discoloration of teeth and dental restorations.^13^ Electronic nicotine delivery systems (ENDS) is on the rise in an attempt to produce smoking cessation in communities. However, it is acting as a gateway for young individuals in becoming regular users.^14^ As a result, it has the potential to become an epidemic.^15^ A common known side effect of ENDS is the discoloration of teeth,^11^ however its effect on oral restorations is not reported. CIELab system was employed for discoloration assessment, as it relates to clinical significance and compares difference in measurements to subjective observations.^16^ Based on this system, a visually perceptable color change is considered to be above ΔE<2.5,^17^ however a ΔE of < 3.7 can go clinically undetected.^18^ The spectrophotometer was used to measure the shade/color difference by a single operator (intra-examiner reliability, kappa=0.85) to prevent operator bias.

In the present study, the ceramic discoloration mean ΔE in both CS and ENDS groups ranged from 2.36 to 2.42. These numbers are comparatively lower in ceramic samples than previous studies, this is attributed to storage of samples in distilled water after exposure.^19^,^20^ Interestingly the change in color for CS and ENDS was comparable (p>0.05). In a recent study by Pintado-Palomino et al., exposure of teeth to ENDS aerosol resulted in ΔE range from 1.9 to 4.6, these findings are similar to the present study in relation to ceramic specimens.^11^ Cigarette smoking results in combustion of elements resulting in release of metals like arsenic, lead and cadmium along with dark components of smoke. These elements deposit on the surface, resulting in discoloration.^14^ By contrast ENDS avoids combustion, however the absorbed e-liquid produces an aerosol by contacting the heating coil, which contains metals like copper, lead and nickel.^14^ These could possibly be a cause of discoloration in samples exposed to ENDS aerosol. Another explanation for these findings is that the e-liquid used in the study was translucent xanthic yellow in color. As yellow color influences the b* at sample post exposure, therefore Δb for ENDS group is higher (1.13 ± 0.3), resulting in a ΔE comparable to CS samples. Therefore, the authors suggest that the color of aerosol can have a significant influence on the discoloring effect and further studies are recommended to assess the influence of different pigmented flavored ENDS aerosols on discoloration.

Discoloration of composite samples showed ΔE values of 42.87 and 46.86 for CS and ENDS specimens respectively. ΔE in excess of 30 has been shown in a previous study with composite materials on exposure to CS.^5^ Although statistically comparable (p>0.05), samples exposed to ENDS aerosol showed slightly higher ΔE compared to CS group. This could be attributed to the nano-particles released in the aerosol of ENDS containing heavy metal elements with pigments and coloring agents.^21^ A myriad of factors influence discoloration of composite resin including, resin type, filler, surface morphology and temperature change.^22^ The higher discoloration values in comparison to previous studies, are attributed to the water sorption and hydrophilicity of resin like Bis-GMA and Bis-EMA (composite used in the present study) causing uptake of water from matrix and staining.^23^ In addition, in the present study, composite specimen surface were not highly polished and were flattened. As studies have shown polishing and texture to influence discoloration of resin composites, this could have resulted in the higher discoloration of composite samples in the present study.^24^

From a clinical perspective, discoloration outcomes for dental restorations, from ENDS use are similar to cigarette smoking; this must be communicated to the patients in the form of patient education. In addition, ENDS use is a contributing risk factor similar to cigarette smoking in oral discoloration. However, study findings should be interpreted in light of certain limitations. The amount of liquid and cigarette consumption for each puff of ENDS and cigarette is not similar, which may influence study outcomes.^11^ In addition, the parameters like, number of puffs, their duration and puff intervals among ENDS and conventional cigarette smoking are difficult to standardize in an in-vitro experiment. Furthermore, the amount of aerosol delivered by any ENDS device differs, therefore the outcomes of the present study should be limited to the device used in the present study (SMOK – Alien).

## CONCLUSION

Aerosol from Electronic nicotine delivery systems (ENDS) showed similar discoloration levels as cigarette smoking (CS). The level of discoloration for ceramic samples for both ENDS and CS was below clinically perceptible levels (Mean ΔE < 2.5). Discoloration of composite resin due to CS and ENDS was visually perceptible (Mean ΔE > 4.0).

### Authors’ Contribution

**FV:** Data collection, study design, manuscript writing, final manuscript approval.

**TA:** Data collection, study design, manuscript drafting, data analysis, funding and equipment, manuscript approval.

**AAs, AAn and OA:** Specimen design and preparation, Data collection, manuscript approval and data interpretation.

**TAM and RA:** Data collection, smoking chamber assembly, writing, revise, editing, final manuscript approval, table and figure designing.

As per the ICMJE authorship requirement, all authors are responsible and accountable for the accuracy or integrity of the work.
